# Supplementary material for: The genome sequence of an ichneumonid wasp, Rhimphoctona ( Xylophylax) megacephalus (Gravenhorst, 1829)
Source: Wellcome Open Res. 2024 Aug 14;9:472. [Version 1] doi: 10.12688/wellcomeopenres.22865.1 (PMC11474148; doi:10.12688/wellcomeopenres.22865.1)
Supplement: Peer review history

# Reviewer report: Reviewer response for version 1

**DOI:** 10.21956/wellcomeopenres.25178.r97325

**Published:** 2024-10-14

**Authors:** Ligia Benavides

**Affiliations:** 1 Harvard University, Cambridge, USA

Boparai *et.al* provide a note on the genome of R. megacephalus, a species of wasp in the family Ichneumonidae, mentioning the relevance of investigating on this particular chromosome-level genome, the first for the genus Rhimphoncta.

 The manuscript is clear and well written. I am not a specialist in the group therefore my comments will focus on the genomic methods.

**Background**

 How big are the specimens of this species?

**Mehtods**

 sample acquisition:

 Remove the words latitude and longitude from the coordinates. 

 How were the specimens killed and stored in the field?

**Assembly:**

 did you filter adapters? how?

 is HiFiASM, not Hifiasm

 also, what version of HiFiASM was used?

 Please add in the text versions of the software used. Although now I saw you have them in the table.

 It would be good a final short comment or conclusion about the relevance of having this particular genome sequenced.

Are sufficient details of methods and materials provided to allow replication by others?

Yes

Is the rationale for creating the dataset(s) clearly described?

Yes

Are the datasets clearly presented in a useable and accessible format?

Yes

Are the protocols appropriate and is the work technically sound?

Yes

Reviewer Expertise:

My expertise is genomic-level data generation, assembly and curation. I am a taxonomist and evolutionary biologist with focus in leaf litter arachnids.

I confirm that I have read this submission and believe that I have an appropriate level of expertise to confirm that it is of an acceptable scientific standard.

# Reviewer report: Reviewer response for version 1

**DOI:** 10.21956/wellcomeopenres.25178.r98635

**Published:** 2024-09-30

**Authors:** Hsuan-Pu Chen

**Affiliations:** 1 Department of Entomology, National Taiwan University, Taipei, Taiwan

Boparai et al. reported the complete genome sequence of the campoplegine Darwin wasp, *Rhimphoctona (Xylophylax) megacephalus*. The paper provides background information, including the biology, faunistic data, morphological characters, phylogenetic position, and phenology of the genus *Rhimphoctona *and the target species. Additionally, it offers statistics for evaluating the assembly of genome sequence.

 In general, the manuscript is well-written in fluent English with sufficient content in both the introduction and genome description. As I am not a specialist in genome sequencing/assembling techniques or bioinformatics analysis, my comments are limited to minor suggestions regarding the background information mainly and some text corrections:

1. In the fifth paragraph of the Background:
  “In their study on parasitoids of saproxylic beetles, Hilszczański *et al*. (2005) (2005) found snags, in particular, hosted a different assemblage of species from other types of dead wood that were tested in the study, with *Rhimphoctona* species abundant and exclusively trapped in the snags created for the study.”
  There is an error for the citation with duplicated year: Hilszczański *et al*. (2005) "(2005)".
2. In Table 2, the percentage of the assembly mapped to chromosomes is 93.5%, which does not reach the benchmark of ≥95%. Similarly, the BUSCO score is C: 94.5% (Benchmark: C ≥ 95%). Although these statistics are numerically close to the benchmark, I am curious about how professionals interpret this data that is not reaching the benchmark when evaluating genome assemblies.

I only have minor comments as mentioned above, and I have no further questions regarding the basic information on the wasps, the section that falls within my expertise. Therefore, I recommend accepting this manuscript after minor revisions.

Are sufficient details of methods and materials provided to allow replication by others?

Yes

Is the rationale for creating the dataset(s) clearly described?

Yes

Are the datasets clearly presented in a useable and accessible format?

Yes

Are the protocols appropriate and is the work technically sound?

Yes

Reviewer Expertise:

I am a taxonomist and systematist specializing in Darwin wasps but have limited knowledge of genome sequencing and assembly. Therefore, my comments are restricted to minor suggestions concerning the background information and some text corrections in this manuscript.

I confirm that I have read this submission and believe that I have an appropriate level of expertise to confirm that it is of an acceptable scientific standard.
